# Evaluation of raw and processed Phellodendri Chinensis Cortex using the quality marker analysis strategy by UHPLC-Q-Orbitrap MS and multivariate statistical analysis

**DOI:** 10.3389/fchem.2023.1223865

**Published:** 2023-07-31

**Authors:** Wang Wang, Xuqin Shi, Guoxue Zhu

**Affiliations:** ^1^ Department of Neurology, Nanjing Hospital of Chinese Medicine Affiliated to Nanjing University of Chinese Medicine, Nanjing University of Chinese Medicine, Nanjing, Jiangsu, China; ^2^ School of Medicine and Holistic Integrative Medicine, Nanjing University of Chinese Medicine, Nanjing, Jiangsu, China; ^3^ School of Artificial Intelligence and Information Technology, Nanjing University of Chinese Medicine, Nanjing, Jiangsu, China

**Keywords:** Phellodendri Chinensis Cortex, raw and processed materials, UHPLC-Q-Orbitrap MS, quality marker analysis strategy, multivariate statistical analysis

## Abstract

**Introduction:** Phellodendri Chinensis Cortex is a necessary part of healthcare for its significant clinical efficacy. Raw and processed Phellodendri Chinensis Cortex is both documented in the Chinese Pharmacopoeia (2015). After processing, the therapeutic effects are believed to differ according to traditional Chinese medicine theories. However, the chemical mechanism responsible for this processing, according to traditional Chinese medicine theories, is still not clear.

**Methods:** In this study, the therapeutic effects of various ions were examined based on traditional Chinese medicine theories by ultra-high performance liquid chromatography-hybrid quadrupole-Orbitrap mass spectrometry (UHPLC-Q-Orbitrap MS) coupled with multivariate statistical analysis, such as principal component analysis (PCA) and orthogonal partial least squares discriminant analysis (OPLS-DA), to comprehensively compare the differences between raw and processed Phellodendri Chinensis Cortex for the first time.

**Results:** A total of 48 compounds were screened, out and 10 of them simultaneously transformed with significant variation in processed products compared with raw materials. It was illustrated that the contents of berberine, palmatine, jatrorrhizine, magnoflorine, menisperine, phellodendrine, tetrahydrojatrorrhizine, and tetrahydropalmatine decreased, while the compounds of berberrubine and fernloylquinic acid methyl ester newly appeared in processed herbs. This is likely to be related to the conversion of ingredients during processing.

**Discussion:** Altogether, the fact that quality markers have been successfully identified to differentiate processed Phellodendri Chinensis Cortex from raw materials suggests that this approach could be used for the investigation of chemical transformation mechanisms involved in the processing of herbal medicine.

## 1 Introduction

Chinese medicine is notably different from Western medicine by its unique pharmaceutical skill in processing Chinese crude drugs. Proper processing of traditional Chinese medicine (TCM) can reduce toxicity or side effects, enhance therapeutic effects, and modify properties by altering the chemical constituents (Cheng et al., 2011). Although some TCM remedies, such as Radix Phytolaccae (Gong et al., 2013), Radix Aconiti Lateralis Preparata (Singhuber et al., 2009), and Semen Strychni (Cai et al., 1996), have been found to have serious toxic side effects, they can be used safely after the toxic elements are eliminated through processing. However, some processing methods can bring harmful effects to our health, such as sulfur-fumigation treatment could cause chemical transformation of original bioactive components and alter bioactivities, or even toxicities (Li et al., 2012). Previously, there has been limited focus from researchers on processed materials, particularly regarding the variances in chemical compositions between raw materials and processed products. Controlling the quality of processed products is an essential and pressing requirement to ensure their safety and effectiveness, given the variations in chemical compositions that can occur between raw materials and the final products, as highlighted by researchers in the field of processed materials.

Phellodendri Chinensis Cortex, which is a TCM, known as “Huangbo,” is prepared from the dried bark of Phellodendron chinense Schneid, has the efficacy of removing damp heat and relieving consumptive fever, and is also utilized in curing dysentery, diarrhea, jaundice, and other syndromes for more than 1000 years in China (Ren et al., 2007). Phellodendri Chinensis Cortex is documented in Chinese Pharmacopoeia (Chen et al., 2015) in three forms, namely, raw (“shengpian” in Chinese, HB), salted products (“yanzhi” in Chinese, YHB), and charring pieces (“tanzhi” in Chinese, THB). As for the clinical application of Phellodendri Chinensis Cortex in China, the raw materials and salted products are mainly applied to diarrhea and jaundice, while the charring pieces are usually utilized for hemostasis (Meng et al., 2004). To date, many studies have been conducted so far on the quality assurance of raw herbs, such as quantitative analysis (Price et al., 2018), chromatographic fingerprinting (Zhang et al., 2018), and extracting techniques (Wang et al., 2015a). In contrast, there has been no research conducted on the quality assurance of processed herbs despite the importance of ensuring their safety and efficacy. The scarcity of chemical data makes it challenging to identify appropriate characteristic compounds that can serve as markers for ensuring the quality control of processed herbs. Therefore, there is a requirement for an efficient and consistent method to identify the components that undergo transformation during processing.

A recently developed technique that utilizes hyphenation is UHPLC-Q-Orbitrap MS. The advancements in precise mass measurements, enhanced retention time reproducibility, heightened sensitivity, superior chromatographic resolution, and accelerated operation speed (İsmail et al., 2018) have transformed UHPLC-Q-Orbitrap MS into an influential instrument for conducting accurate and precise metabolomics studies (Qi et al., 2018). Additionally, the accurate mass values generated using the Orbitrap MS instrument enable the identification of candidate empirical formulas, significantly reducing the potential number of structures for putative compounds compared to alternative methods (Zuo et al., 2017). In recent years, UHPLC-Q-Orbitrap MS has gained popularity as a rapid method for conducting global chemical profiling of TCMs (Hu et al., 2017).

**FIGURE 1:**
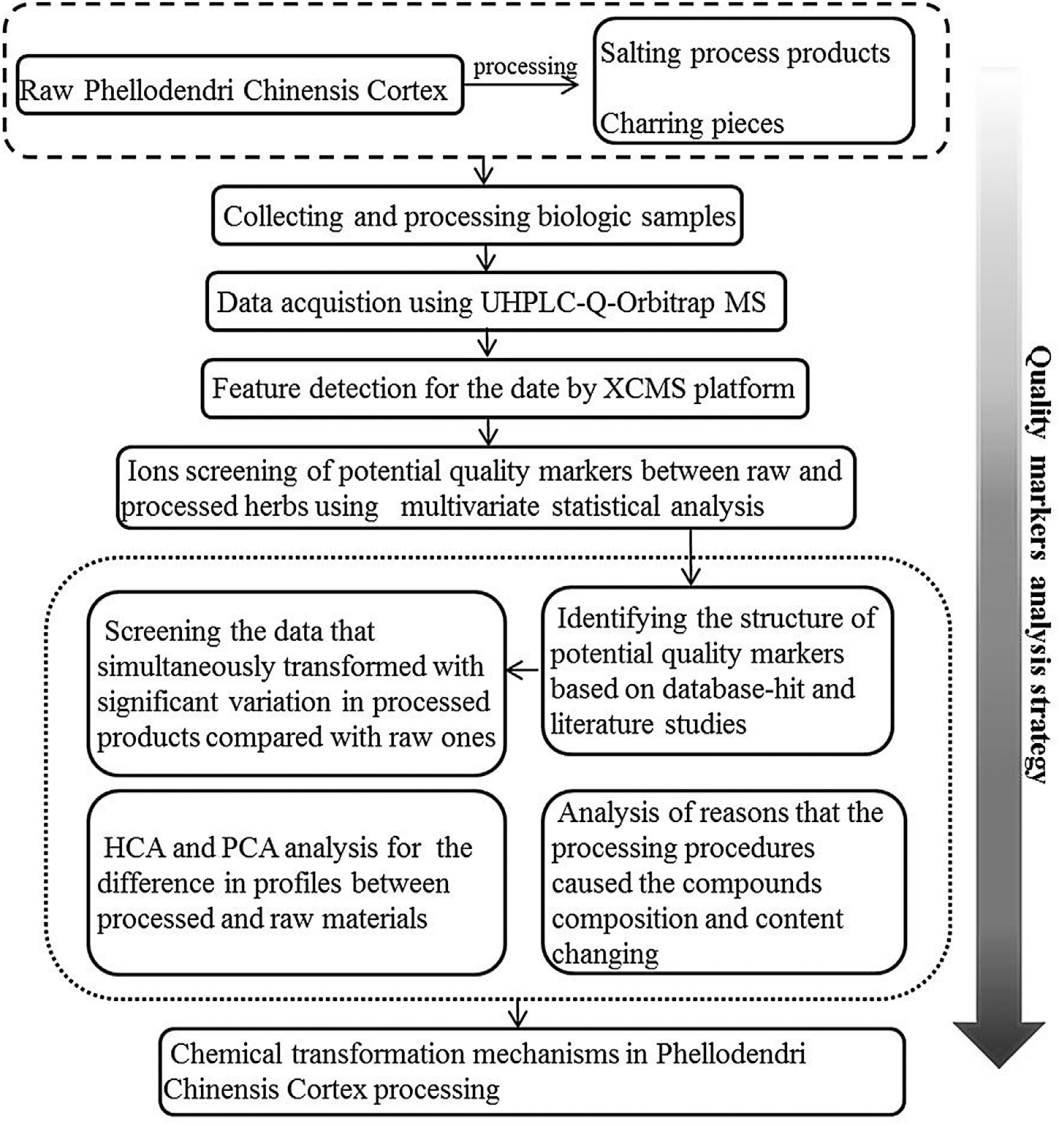
Schematic representation of the quality marker analysis strategy utilized for HB.

In this study, using Phellodendri Chinensis Cortex as an example, the quality marker analysis strategy (Figure 1) based on UHPLC-Q-Orbitrap MS and multivariate statistical analysis is proposed for rapid identification and discrimination of raw and processed samples. To detect quality indicators, the UHPLC-Q-Orbitrap MS instrument was utilized to acquire metabolic profiles from both unprocessed and processed samples. Pairwise comparisons between HB vs. YHB and HB vs. THB were performed by metabolic profiles and multivariate statistical analysis including PCA, OPLS-DA, and hierarchical cluster analysis (HCA). The compounds that show correlation with these ions are likely to be the transformed compounds induced by processing and could be considered potential quality indicators for distinguishing between raw and processed herbs. The numerical outcomes indicate that this method of investigation could be an advantageous approach in discovering the processing mechanisms of different herbs.

## 2 Experimental methods

### 2.1 Reagents and herbal materials

The HPLC-grade acetonitrile and formic acid were bought from Merck (Darmstadt, Germany). Methanol of HPLC grade was acquired from Fisher Scientific (Pittsburgh, United States). The Millipore-Q water purification system (Bedford, United States) was utilized to produce ultra-high purity water. The remaining reagents employed were of analytical grade.

Six batches of raw Phellodendri Chinensis Cortex (NO. A1-A6) and processed materials, including six batches of salted products (NO. C1–C6) and six batches of charring pieces (NO. D1–D6), were purchased from Kangmei Pharmaceutical Co., Ltd. (Puning, China).

### 2.2 Sample preparation

Various samples of Phellodendri Chinensis Cortex were ground into a uniform size and filtered through a No. 40 mesh. The precisely measured powder (0.1 g) was exposed to ultrasonication using a KQ-3200E sonicator (Kunshan Ultrasonic Instruments Co., Ltd.) at an ambient temperature for 1 h to extract it with a mixture of 10 mL methanol and water (75:25, v/v). The extracts underwent filtration and were then diluted to the desired volume using a 10-mL volumetric flask containing 75% methanol. After filtration through a 0.22-μm membrane, a 5-μL aliquot of the solution was injected for UHPLC-Q-Orbitrap MS analysis.

### 2.3 LC-MS/MS conditions

Q-Exactive™ Plus Orbitrap (Thermo Fisher Scientific, San Jose, United States), connected to the UHPLC system consisting of a HESI ion source as the interface, operated under Q-Exactive Tune 2.3 Build 1765. Chromatographic separation analysis was performed with the ACQUITY UHPLC BEH C18 column (100 mm × 2.1 mm, 1.7 μm). The mobile phases, consisting of solvent A (HCOOH/CH_3_CN = 0.2:100) and solvent B (HCOOH/H_2_O = 0.2:100), were employed with a gradient elution approach outlined as follows: the gradient started at 10% A and was raised to 20% A in 3 min. It was then gradually raised to 60% A over a period of 2 min. Then, it was further raised to 80% A in 2 min and held at that level for 2 min. The sample was injected with a volume of 5 μL, and the flow rate was adjusted to 0.3 mL/min.

For both the negative and positive switch modes, the electrospray ionization interface (HESI-II) was maintained at a voltage of 3200 V. The heater temperature was calibrated to 350 °C, and the heated capillary temperature was fine-tuned to 320 °C. All the screening data were acquired through the utilization of the full MS scan mode in combination with the dd-MS2 mode. The complete range of mass-to-charge (m/z) values from 100 to 1,000 was covered in the acquired spectra during the scan. The resolution of the Orbitrap was adjusted to 70,000 full-width at half-maximum (FWHM) at m/z = 200, enabling the acquisition of approximately three full scan-ddMS2 (data points) per second. The quadrupole served as a broad-spectrum mass filter (m/z 100–1000).

### 2.4 Method validation

The pooled QC sample, which consisted of a mixture of 50 μL from all samples, was utilized to guarantee the reliability of analytical instrumentation and the consistency of the data. To ensure the dependability of analytical equipment and the uniformity of the results, a QC sample was injected again at the start, after every four sample injections, and at the conclusion of the experiment, producing a dataset for monitoring the instrument stability and assessing reproducibility.

### 2.5 Data processing and chemometrics analysis

The potential quality markers for distinguishing and monitoring the quality of both raw and processed Phellodendri Chinensis Cortex samples were identified by analyzing the UHPLC-Q-Orbitrap MS data on all determined samples using SIMCA-P software (13.0 demo version, Umetrics, Sweden). Data from all identified samples were uploaded to the XCMS online platform (https://xcmsonline.scripps.edu) to perform feature detection. According to the information provided, we performed feature detection on multiple sets of samples on the XCMS online platform, such as 5-ppm mass error and 5 and 20 scan time units peak width in feature detection, Obiwarp method and value option in retention time correction, and 0.025 mzwid and 0.5 minfrac in alignment. We select the data that exhibit a relative standard deviation (RSD) below 30% as our final result. The data, consisting of the peak number (t_R_-m/z pair), sample name, and ion intensity in three dimensions, were subjected to PCA and OPLS-DA analysis using SIMCA-P software. The R^2^(Y) and Q^2^(Y) parameters were utilized to evaluate the validity and robustness of the models. The extent of variation in R^2^(Y) plays a critical role in the interpretation of OPLS-DA plots.

First, PCA was utilized to generate the score plots, which represent the samples that were clustered to evaluate the separation of the chemical profiling of raw materials and processed products. Second, the utilization of OPLS-DA was employed to discover potential quality markers that play a significant role in discriminating between raw and processed products. Then, the data that *p* < 0.01 and variable importance in projection (VIP) > 1 based on OPLS-DA combined with a variance cross-validation test (CV-ANOVA) were screened out and regarded as potential quality markers among raw materials and processed products. Finally, HCA was performed to evaluate the variation in raw and processed materials using SPSS software 20.0 version (SPSS Inc., Chicago, United States) based on the characteristic of the peaks obtained from UHPLC-Q-Orbitrap MS profiles.

## 3 Results and discussion

### 3.1 Results and discussion

The extraction parameters were fine-tuned in order to increase the number and intensity of the chromatographic peaks. Based on the intensity and quantity of the obtained peaks, this study tested various extracting solvents, including methanol solvents with different concentrations (50%, 75%, and 100%) coupled with varying ultrasonic extraction times (45 min, 60 min, and 75 min). The findings indicate that ultrasonic extraction for 30 min using 75% methanol was the most suitable approach.

### 3.2 Optimization of chromatographic and mass spectrometric conditions

To obtain the global chemical profiling of the herbal medicine, MS spectra were acquired in both positive and negative ion modes. Meanwhile, mobile phase, elution conditions, and mass spectrometric conditions on the intensity of the total ion current of the sample solution were examined. By comparison, 0.2% formic acid aqueous solution and acetonitrile were selected to enhance ionization and improve the peak shape efficiency, and MS spectra were obtained in both positive and negative ion modes. The effects of capillary voltage, sample cone voltage, and desolvation temperature were performed with the aim to enhance the intensity and quantity of the total ion chromatograms (TICs).

### 3.3 Method validation

The ion intensities of ten characteristic peaks were calculated, which were obtained from the TICs of the QC samples. The precision, repeatability, and stability of the method were then evaluated. Measurements from a single sample solution stored at room temperature for 0, 2, 4, 8, 12, 16, and 24 h were used to assess the stability of the sample. The results demonstrated that all of signal intensity variations were not more than 5%. QC samples were employed during the batch procedure to assure the reliability of the quality marker strategy. The fluctuations in the magnitude of the variables were within a 20% range. The data gathered for every (t_R_)-m/z pair are documented in the list. Therefore, the results indicated that the samples and measurements were stable and under control.

### 3.4 HCA for the identification of raw and processed materials

**FIGURE 2:**
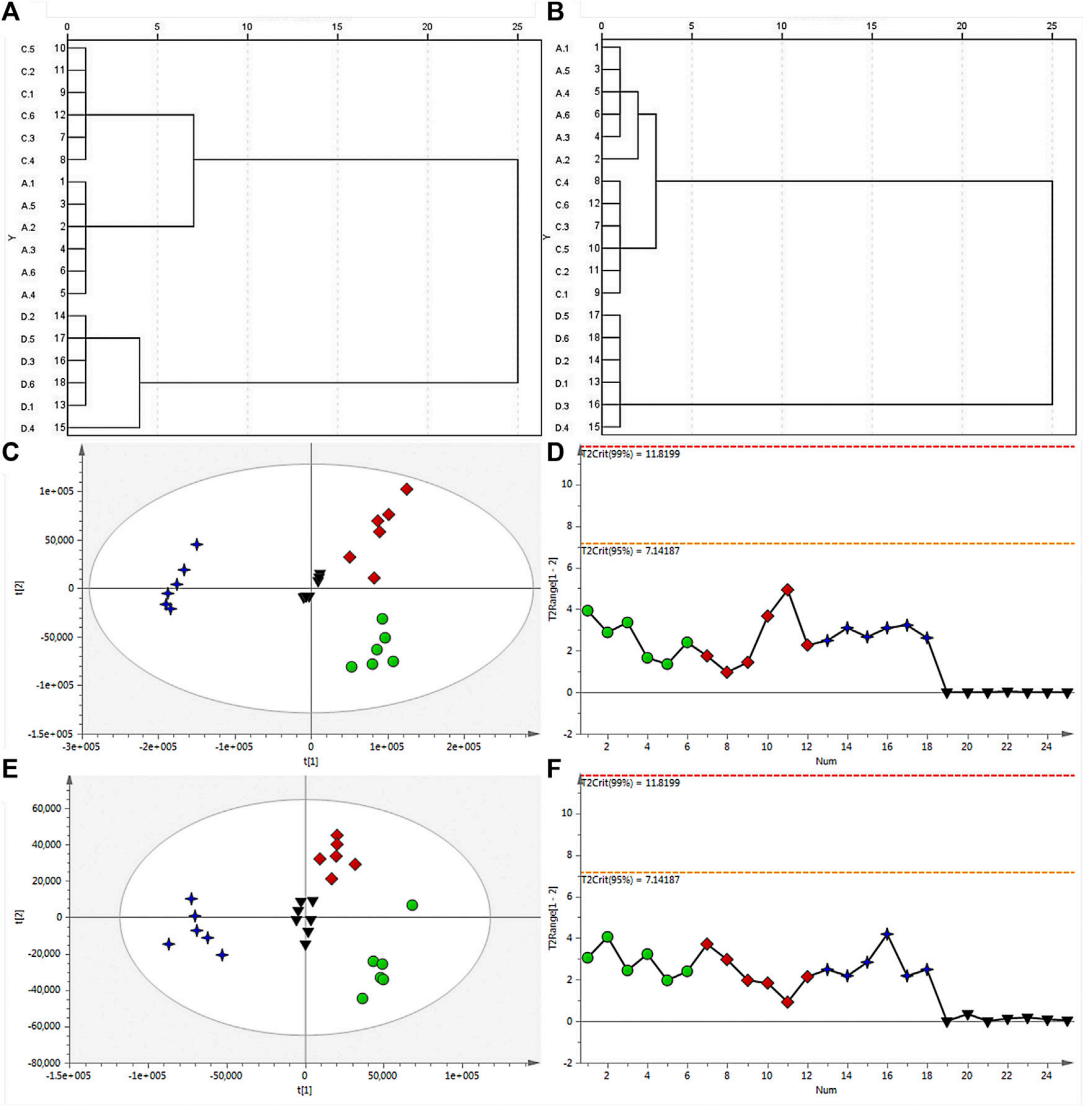
Dendrogram of HCA for raw and processed materials of HB in the positive **(A)** and negative ion modes **(B)**. **(C)** PCA score plot of all analyzed samples in the positive ion mode with the statistical parameters (R^2^X = 0.882, Q^2^ = 0.764), and **(D)** corresponding Hotelling’s T2 range plot showing the sample type to detect any trends in QC and test samples. **(E)** PCA score plot of all analyzed samples in the negative ion mode with the statistical parameters (R^2^X = 0.733, Q^2^ = 0.622), and **(F)** corresponding Hotelling’s T2 range plot. HB (green dot), YHB (red diamond), and THB (blue 4-point star) samples, as well as QC samples (black inverted triangle), are shown.

HCA serves as a method for arranging a complicated collection of observations into discrete clusters of subjects that share similarities in specific characteristics and are mutually exclusive from one another (Zhou et al., 2015; Ulrich et al., 2017). HCA operates on the principle of grouping n samples into distinct classes based on their similarity to one another. In this study, the outcome of the study indicated distinct classification of the samples into three categories. It illustrated that HB and YHB clustered into one group first and then clustered with THB. This result showed that the processing procedures could change the compositions and contents of compounds, while the batch of charring pieces changes considerable in chemical constituents than salted products compared with raw materials. In addition, this is consistent with the analysis of PCA between raw and processed materials. HCA results in positive and negative modes are shown in Figures 2A, B.

### 3.5 Multivariate analysis of chemical profiling

Before analysis, Q-Exactive Tune 2.3 Build 1765 was utilized to collect the raw data. The representative TICs of HB, YHB, and THB both positive and negative ion modes are shown in Supplementary Figure S1. After extraction and filtering, the XCMS platform can reveal a number of features to these three groups of data. Unsupervised PCA and supervised OPLS-DA were carried out to assess the distinction between raw and processed Phellodendri Chinensis Cortex samples. Upon application of Pareto scaling with mean-centering, the data were represented as a PCA score plot (Figures 2C, E). It shows that the determined samples clearly clustered into three groups, i.e., the raw, salted products, and charring pieces, indicating that the processing procedures caused changes in the composition and/or content of compounds in Phellodendri Chinensis Cortex. In the PCA score plot, the X-axis and Y-axis represent the variance associated with PC 1 and 2, respectively. The sample category results were quite similar whether in positive or negative ion mode, which hinted that YHB and HB samples located more tightly further illuminate the charring piece change considerable in chemical constituents than salted products compared with raw materials. Hotelling’s T2 range plots were used to evaluate the homogeneity of sample sets and to detect potential outliers. In order to scrutinize the quality of the data and the impact of the run order on both the test and QC samples in positive (Figure 2C) and negative ion modes (Figure 2E), the corresponding Hotelling’s T2 range plot was employed, as shown in Figures 2D–F, with significance limits of 95% and 99%. Any value larger than the yellow limit (0.05) should be viewed with suspicion, while any value larger than the red limit (0.01) is considered a severe outlier. The outcome revealed the absence of outliers and any time-dependent effects on the identified ions.

An S-plot was generated through OPLS-DA to identify potential quality markers (Supplementary Figure S2). Supplementary Figure S2A–D shows ion pairs t_R_-m/z, with each variable point represented. Variable contribution is plotted on the X-axis, and variable confidence is plotted on the Y-axis. Greater contribution to classification is indicated by a greater distance from the origin. Therefore, the variables located at the sharp ends of the “S” shape were identified as potential characteristic chemical markers, making the greatest contribution to discriminating the two groups. To simplify the data analysis, additional filtering procedures were conducted based on one-way ANOVA (*p* < 0.05) and VIP value (VIP > 1) analysis.

### 3.6 Rapid identification of quality markers

**FIGURE 3:**
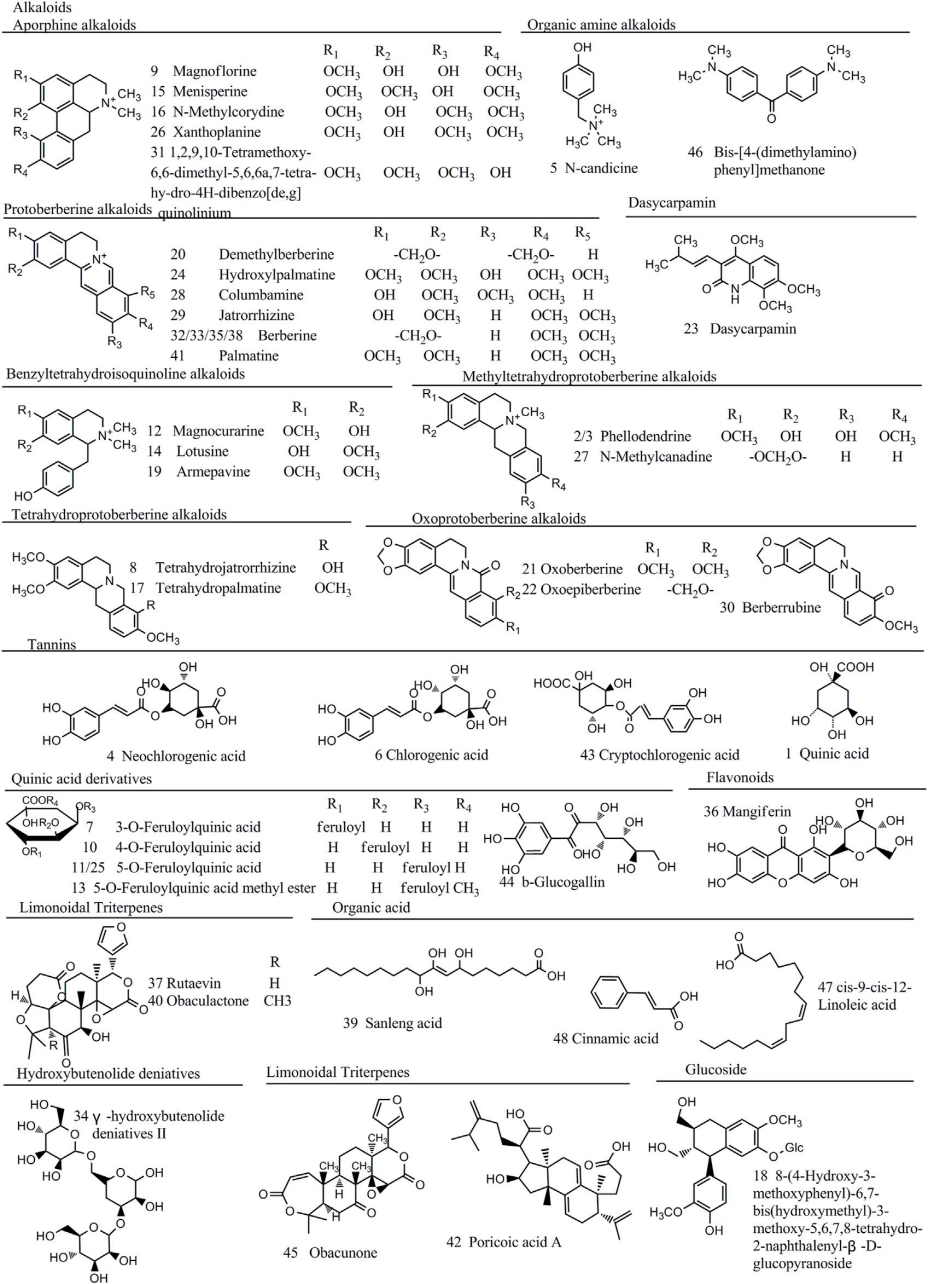
Chemical structure of 48 quality control marker compounds identified in raw and processed materials of HB.

To assign characteristic components, accurate mass was obtained to calculate the product of several possible molecular formulas (within a mass error of 10 ppm) by Xcalibur 3.0.63 software. Then, we obtained the possible fragments of molecular candidates and compared with the proposed probable fragmentation patterns through MS2 mass spectrometry. At last, a total of 48 compounds were screened out and characterized, which were discriminant variables in HB, YHB, and THB, including alkaloids, tannins, quinic acids, and limonoid triterpenes. The identification information and structural formulas are summarized in Figure 3 and Supplementary Table S1.

#### 3.6.1 Identification of alkaloids

A total of 24 alkaloids including protoberberine alkaloids, tetrahydroprotoberberine alkaloids, aporphine alkaloids, and benzyltetrahydroisoquinoline alkaloids were preliminarily identified in the positive ion mode.

**FIGURE 4:**
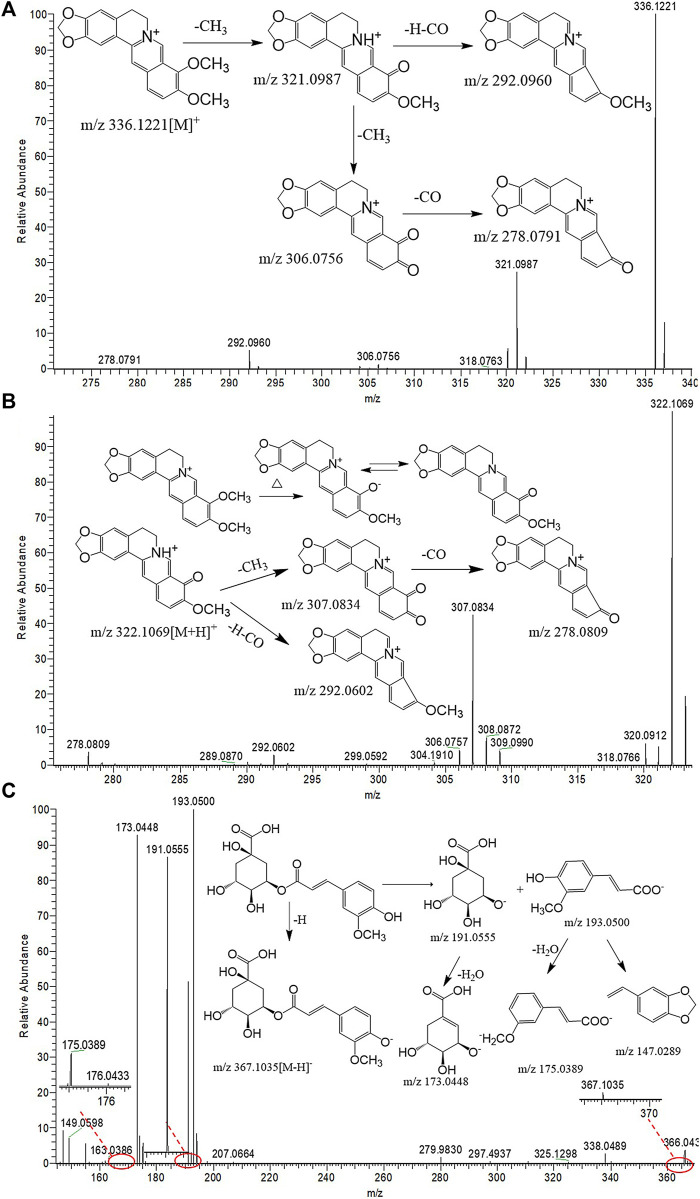
UHPLC-Q-Orbitrap MS spectral and proposed fragmentation pathways of berberine **(A)**, berberrubine **(B)**, and 3-O-feruloylquinic acid **(C)**.

Nine protoberberine alkaloids including compounds 20, 24, 28, 29, 32, 33, 35, 38, and 41 were selected for extraction ion analysis. Compound 32 exhibited the MS characteristic of m/z 336.1221 [M]^+^ and calculated to be C_20_H_18_NO_4_. The generated fragment ions, as shown in Figure 4A, at m/z 321.0987 [M-CH_3_]^+^, 306.0756 [M-2CH_3_]^+^, 292.0960 [M-CH_3_-H-CO]^+^, and 278.0791 [M-2CH_3_-CO]^+^ were observed in their MS^2^ fragmentations. The accurate mass and fragment ions conform with berberine, as described in literature studies (Xian et al., 2014), which was a known compound found in HB. Following this method, compound 20 generated an [M]^+^ ion at m/z 324.1223 and 12 Da lower than the mass of compound 32. Tentative identification of the compound was performed as demethylberberine. Compound 41 produced [M]^+^ ions at m/z 368.1488 and 352.1169, and then exhibited fragments at 337.0938 [M-CH_3_]^+^, 308.0916 [M-CH_4_-CO]^+^, and 292.0981 [M-4CH_3_]^+^. Compound 24 was 16 Da higher than the mass of compound 41. Combined with the literature (Wu et al., 2005), compounds 24 and 41 were identical as palmatine hydroxyl and palmatine, respectively. Compounds 28 and 29 were considered to be columbamine and jatrorrhizine by the same way, respectively.

Compounds 9, 15, 16, 26, and 31 showed the characteristics MS [M]^+^ of m/z were 342.1694, 356.1850, 356.1849, 356.1849, and 370.2007, respectively. In MS^2^, fragment ions were produced at [M-(CH_3_)_2_NH]^+^, [M-(CH_3_)_2_NH-CH_3_OH]^+^, and [M-(CH_3_)_2_NH-CH_3_OH]^+^. The observed findings are believed to exhibit the typical properties of aphorphine alkaloids. As shown in Zhao et al. (2010), compound 9 showed the same fragment pathways as magnoflorine. Moreover, comparing with the retention time, characteristic MS and fragments with standards, compounds 15, 16, 26, and 31 were identical as menisperine, N-methylcorydine, xanthoplanine, and 1,2,9,10-tetramethoxy-6,6- dimethyl-5,6,6a,7-tetrahydro-4H-dibenzo [de,g] quinolinium, respectively (Liu et al., 2015).

Compounds 21 produced [M]^+^ ion at m/z 352.1172 and 16 Da higher than berberine. The fragment ions at 337.0936 [M-CH_3_]^+^, 322.1155 [M-2CH_3_]^+^, and 308.1354 [M-CH_3_-CO-H]^+^ were similar with berberine. In addition, comparing with the characteristic MS and fragments with standards, compounds 21, 22, and 30 were identified as oxoberberine, oxoepiberberine, and berberrubine, respectively (Figure 4B).

#### 3.6.2 Identification of tannins

Compounds 4, 6, and 43 generated [M-H]^−^ ion at m/z 353.0879, of which it was found to be C_16_H_18_O_9_, and then showed fragment ion at 191.0553 [M-C_9_H_6_O_3_]^−^ through loss a caffeoyl group, but the substituent group can be seen at C-1, C-3, and C-5. According to the retention time, compound 6 was identified as chlorogenic acid. The isotopic abundance ratio is lower when the substituent group is located at C-1, and compound 4 is identified as neochlorogenic acid. Using this method, compounds 43, 44, and 1 were assigned as cryptochlorogenic acid, b-glucogallin, and quinic acid, respectively.

#### 3.6.3 Identification of quinic acid derivatives

Compounds 7, 10, 11, and 25 exhibited [M-H]^−^ and fragment ions at the negative mode in the full scan mode. These compounds produced [M-H]^-^ ions at m/z 367.1032, and the quasimolecular ion lose a feruloyl group (176 Da) to generate a characteristic fragment ion 191.0554 [M-H-C_10_H_9_O_4_]^−^. It conforms with quinic acid derivatives, which is already found in HB. Then, it is tentatively identified as feruloylquinic acid in line with the accurate mass values and fragments, and the feruloyl group can be substituted at C-3, C-4, and C-5. According to the retention time and literature (Sun et al., 2016), tentative identification of compound 7 as 3-O-feruloylquinic acid and the fragmentation pathways are proposed in Figure 4C. Compounds 10, 11, and 25 were assigned as 4-O-feruloylquinic acid, 5-O-feruloylquinic acid, and the isomer of feruloylquinic acid, respectively. Before the peak of berberine, processed materials showed a new compound which produced the [M-H]^−^ ion at m/z 381.1195, the H atom of carboxyl in fernloylquinic acid substituted by the methyl group to become fernloylquinic acid ester. Then, compound 13 is identified as the 3/4/5-O-fernloylquinic acid methyl ester.

#### 3.6.4 Identification of other compounds

Compound 5 produced a molecular ion at m/z 180.1380, of which the accurate molecular formula was C_11_H_18_NO. The fragment ion 121.0648 [M-C_3_H_10_N]^+^ appeared in MS^2^ and considered to be N-candicine. Compound 46 showed the 313.2386 [M + HCOO]^−^ ion in MS and found the 148.0506 [M + HCOO-CH_3_-C_7_H_8_N]^−^ ion in MS^2^. It is apparently a symmetry structure and is identified as bis-[4-(dimethylamino) phenyl] methanone. Compound 23 (m/z 349.1525) generated fragment ions at 334.0684 [M + HCOO-CH_3_]^−^, 286.0726 [M-H_2_O]^-^, and 175.0390 [M-H-CO]^−^ as major MS. It was considered as dasycarpamin based on the previous report (Wang et al., 2013).

Using full scan methods in the negative mode, two limonoid triterpenes were tentatively identified from the HB samples. Compound 37 contains the fragment ions at m/z 423.1819, m/z 411.2003, and m/z 327.2176. The results conform with the previous study (Zhang et al., 2018), and the compound is identified as rutaevin. The plausible mechanistic pathway is shown in Supplementary Table S1. Compound 40 showed a similar fragmentation pathway with compound 37 and quasimolecular ions 15 Da higher than it. Thus, compounds 40 and 45 are considered to be obaculactone and obacunone, respectively.

Compounds 39, 47, and 48 were identified as organic acid and possess 329.2333 [M-H]^-^, 279.2312 [M-H]^-^, and 149.0231 [M + H]^+^ ions. According to the mechanistic pathways, shown in Supplementary Table S1, and previous study (Zhao et al., 2008), compounds 39, 47, and 48 were considered to be sanleng acid, cis-9-cis-12-linoleic acid, and cinnamic acid, respectively. Utilizing the aforementioned methods and MS characteristics, mechanistic pathways, and the previous studies (Li et al., 2010; Wang et al., 2013; Wang et al., 2015b; Sun et al., 2016), compounds 18, 34, 36, and 42 were identified as 8-(4-hydroxy-3-methoxyphen-yl)-6,7-bis(hy-droxymethyl)-3-methoxy-5,6,7,8-tetrahydro-2-naphthalenyl-β-D-glucopyranoside, γ-hydroxybutenolide deniatives II, mangiferin, and poricoic acid A, respectively.

### 3.7 Difference between raw and processed Phellodendri Chinensis Cortex

**FIGURE 5:**
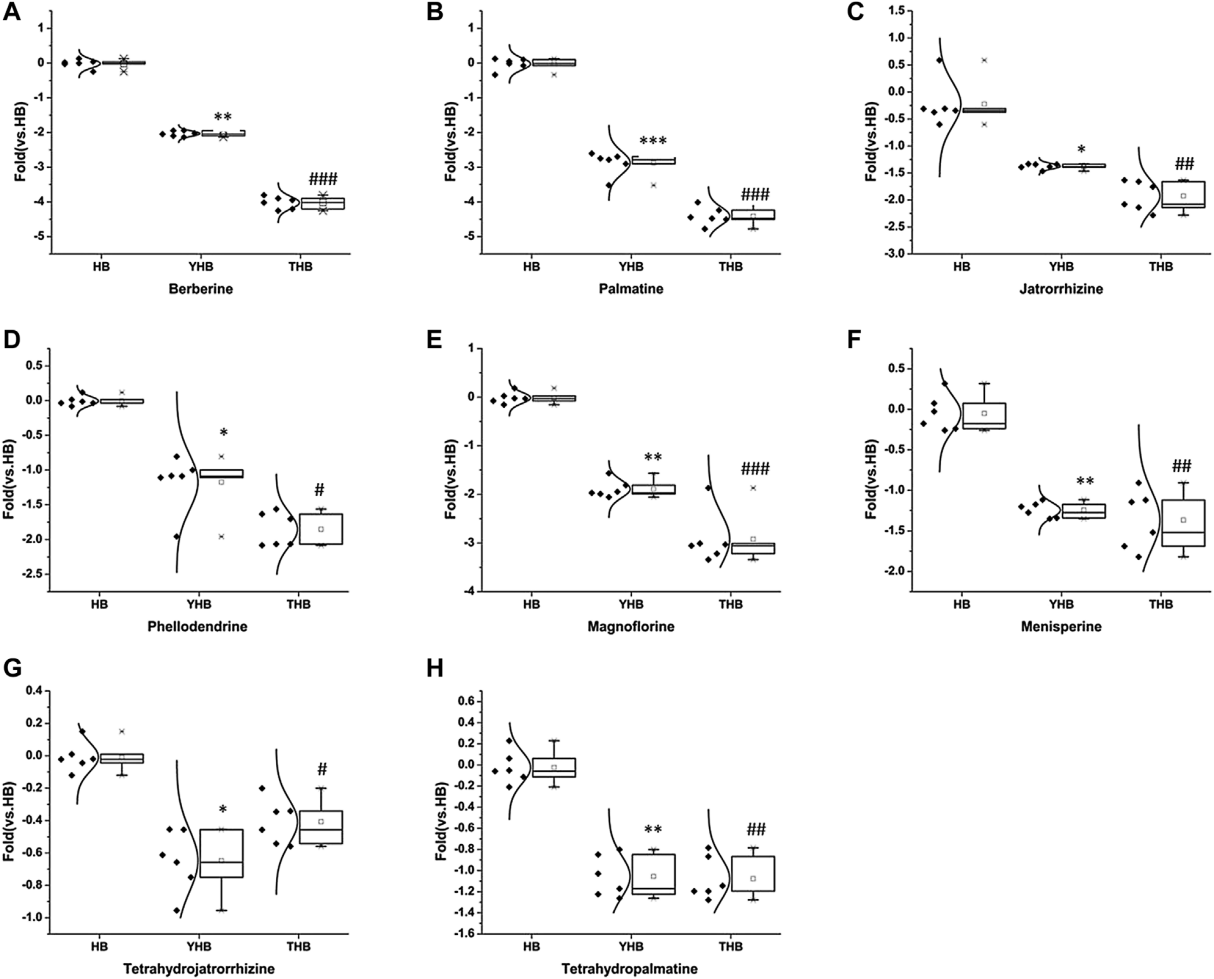
Variations in the trends of the metabolites that are simultaneously transformed in YHB and THB compared with HB. **(A–H)** Variations in the trends of berberine, palmatine, jatrorrhizine, magnoflorine, menisperine, phellodendrine, tetrahydrojatrorrhizine, and tetrahydropalmatine. ***p < 0.001, **p < 0.01, and *p < 0.05 compared with the HB group; ^###^ *p* < 0.001, ^##^ *p* < 0.01, and ^#^ *p* < 0.05 compared with the HB group.

Matching the empirical molecular formula with those of published compounds in Phellodendri Chinensis Cortex allowed for the identification of the identities of potential markers and the major peaks detected in HB, YHB, and THB. A total of 48 compounds were screened out between the two group of HB vs. YHB and HB vs. THB. Compared with the HB group, 10 of them simultaneously transformed with significant variation in YHB and THB compared with HB. Figure 5 shows the boxplot analysis of metabolite levels in HB, YHB, and THB, and other two compounds newly appeared in processed herbs. It is obvious that the contents of berberine, palmatine, jatrorrhizine, magnoflorine, menisperine, phellodendrine, tetrahydrojatrorrhizine, and tetrahydropalmatine decreased, while the compounds of berberrubine and fernloylquinic acid methyl ester newly appeared in processed herbs. This is likely to be related to the conversion of ingredients during processing. As during the process of heating, berberine loses a methyl group to become ionic berberrubine; then, isomerization ketone forms. The mechanistic pathway of berberrubine and the transformation process is shown in Figure 4B. Additionally, the H atom of carboxyl in fernloylquinic acid is substituted by a methyl group to become fernloylquinic acid ester.

The contents of active compounds in a herbal drug can undergo significant changes before and after processing, leading to different therapeutic effects, as evident. Furthermore, berberine is recognized for its anti-inflammatory, hepatoprotective, and anti-oxidant effects, among others (Zhang et al., 2014). In addition to berberine, the main protoberberine-type alkaloids also exhibited various biological activities similar to those of berberine (Zhang et al., 2014; Zhao et al., 2016). Magnoflorine has been shown to activate p38 MAPK and Akt signaling, resulting in an increase in the expression of myogenic factors (MyoD and MHC) (Lee et al., 2017; Meng et al., 2018). This activity is believed to be linked to muscle degeneration and pro-inflammatory responses, while berberrubine possess the pharmacological activity of hemostasis (Bao et al., 2009). The results are consistent with the clinical application and have a preliminary study of the herb-processing mechanism.

## 4 Conclusion

The alteration of chemical constituents through processing is a key characteristic of TCM, facilitating its diverse pharmacological effects and clinical applications. This study developed an effective strategy for analyzing quality markers by combining UHPLC-Q-Orbitrap MS and multivariate statistical analysis. The strategy was successfully applied for the global analysis of chemical constituents in HB, YHB, and THB, and was able to differentiate raw from processed herbs. Unlike conventional approaches that require the laborious and time-consuming characterization of numerous components, this novel strategy eliminates the need for duplicative isolation, purification, and identification of identical components in raw and processed herbs. As a result, 48 compounds were screened out between the two groups of HB vs. YHB and HB vs. THB. Compared with the HB group, 10 of them simultaneously transformed with significant variation in YHB and THB compared with HB. Processed products showed significant decrease in the content of alkaloids, which are mainly responsible for the purgative activity of Phellodendri Chinensis Cortex. In conclusion, the identification of quality markers to distinguish processed Phellodendri Chinensis Cortex from raw materials demonstrates the potential of this strategy for studying the chemical transformation mechanisms involved in herb processing.

## Data Availability

The original contributions presented in the study are included in the article/Supplementary Material; further inquiries can be directed to the corresponding author.
