# Supplementary material for: Evaluation of raw and processed Phellodendri Chinensis Cortex using the quality marker analysis strategy by UHPLC-Q-Orbitrap MS and multivariate statistical analysis
Source: Front Chem. 2023 Jul 31;11:1223865. doi: 10.3389/fchem.2023.1223865 (PMC10423935; doi:10.3389/fchem.2023.1223865)
Supplement: Supplementary file 1 [file DataSheet1.DOCX]

Supplementary Material

Evaluation of raw and processed Phellodendri Chinensis Cortex using quality markers analysis strategy by UHPLC-Q-Orbitrap MS and multivariate statistical analysis

Wang Wang^1,2 †^, Xuqin Shi^3†^, Guoxue Zhu ^1*^

^1^ Department of Neurology, Nanjing Hospital of Chinese Medicine affiliated to Nanjing University of Chinese Medicine, Nanjing University of Chinese Medicine, Nanjing, Jiangsu, China;

^2^ School of Medicine & Holistic Integrative Medicine, Nanjing University of Chinese Medicine, Nanjing, Jiangsu, China;

^3^ School of Artificial Intelligence and Information Technology, Nanjing University of Chinese Medicine, Nanjing, Jiangsu, China;

^†^These authors contributed equally to this work

*** Correspondence:**Guoxue Zhu
zgxue0122@njucm.edu.cn

Fig.S1The UHPLC-Q-Orbitrap MS total ion chromatograms of raw materials of *HB* in positive (A) and negative ion mode (B); The UHPLC-Q-Orbitrap MS total ion chromatograms of *YHB* in positive (C) and negative ion mode (D); The UHPLC-Q-Orbitrap MS total ion chromatograms of *THB* in positive (E) and negative ion mode (F)

**A**

**B**

**C**

**D**

**E**

**F**


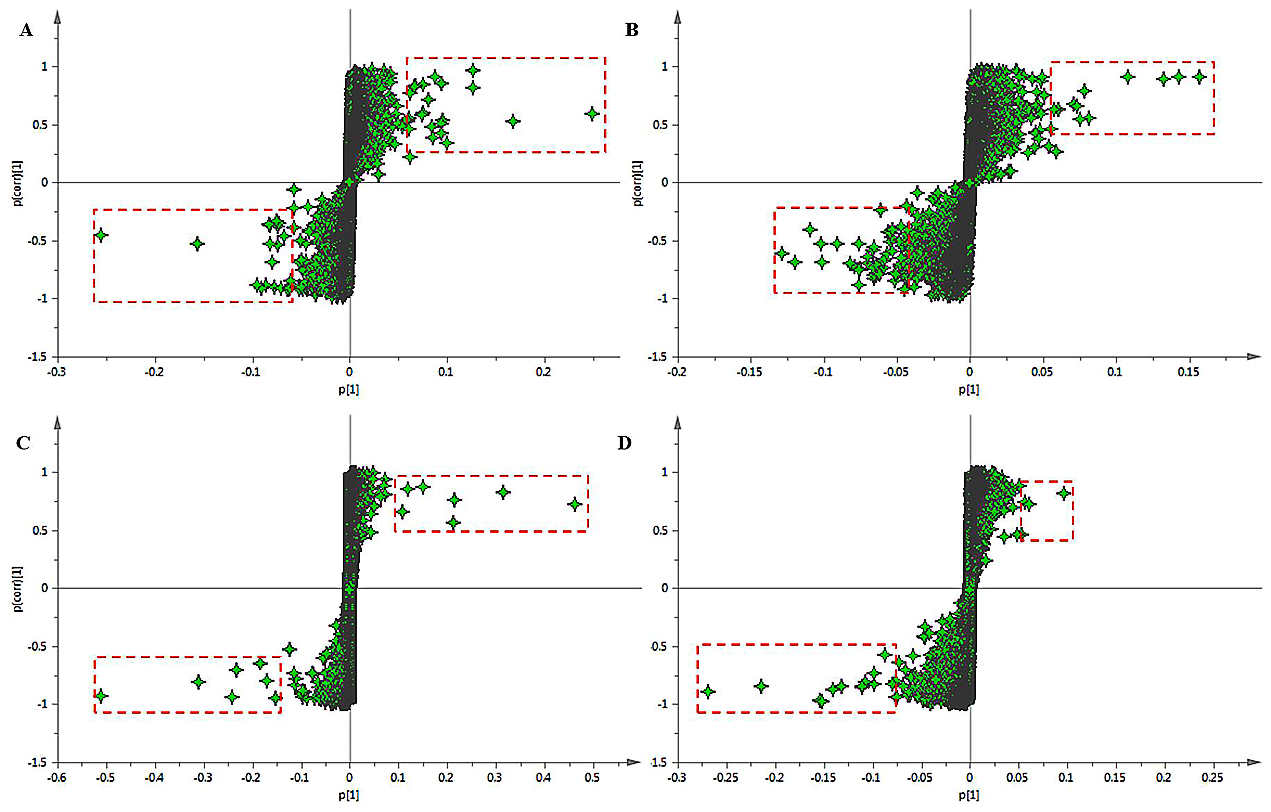


**Fig.S2** S-plot of OPLS-DA model between *HB* and *YHB* in positive ion mode (A) and negative ion mode (B); S-plot of OPLS-DA model between *HB* and *THB* in positive ion mode (C) and negative ion mode (D).

Table S1. Identification of characteristic markers compounds in raw and processed materials of HB

| No. | t_R_  (min) | Formula | Determined mass/Da  (m/z) | | Error/ppm | ESI^-^ | ESI^+^ | Identified compounds |
| --- | --- | --- | --- | --- | --- | --- | --- | --- |
|  |  |  | [M-H]- | [M+H]+ |  |  |  |  |
| 1 | 0.69 | C_7_H_12_O_6_ | 191.0554 |  | -3.645 | 189.0397[M-2H]^−^  173.0324[M-H-H_2_O] ^–^  155.0339[M-H-2H_2_O] − |  | Quinic acid |
| 2 | 0.72 | C_20_H_24_NO_4_ |  | 342.1692 | -3.799 | _ | 297.1118[M-OCH_3_-CH_3_ ]^+^ 192.1016[M-C_9_H_11_O_2_]^+^  177.0784[M-C_9_H_11_O_2_-CH_3_] ^+^ | Phellodendrine |
| 3 | 0.81 | C_20_H_24_NO_4_ |  | 342.1693 | -3.507 | _ | 297.1118[M-OCH_3_-CH_3_ ]^+^ 192.1016[M-C_9_H_11_O_2_]^+^  177.0784[M-C_9_H_11_O_2_-CH_3_] ^+^ | Isomer of Phellodendrine |
| 4 | 0.88 | C_16_H_18_O_9_ | 353.0879 |  | 0.282 | 193.0602[M-C_7_H_11_O_5_]^-^  191.0553[M-C_9_H_6_O_3_]^-^  173.0447[M-C_9_H_6_O_3_-H_2_O]^-^ | _ | Neochlorogenic acid |
| 5 | 0.92 | C_11_H_18_NO |  | 180.1380 | -4.441 | _ | 121.0648[M-C_3_H_10_N]^+^ | N-candicine |
| 6 | 1.97 | C_16_H_18_O_9_ | 353.0879 |  | 0.282 | 193.0602[M-C_7_H_11_O_5_]^-^  191.0554[M-C_9_H_6_O_3_]^-^  173.0445[M-C_9_H_6_O_3_-H_2_O]^-^ |  | Chlorogenic acid |
| 7 | 2.22 | C_17_H_20_O_9_ | 367.1035 |  | 8.728 | 193.0500[M-H-C_7_H_11_O_6_]^−^  191.0555[M-H-C_10_H_9_O_4_]^−^ 175.0389[M-H-C_7_H_11_O_6_-H_2_O]^−^ 173.0448[M-H-C_10_H_9_O_4_-H_2_O]^−^ 147.0289[M-H-C_7_H_11_O_6_-CO_2_-H_2_O]^−^ | _ | 3-O-feruloylquinic acid |
| 8 | 2.80 | C_20_H_23_NO_4_ |  | 342.1694 | -1.759 | _ | 298.0124[M+H-CH_2_CH_2_-CH_3_-H]^+^ 268.0858[M+H-CH_2_CH_2_-CH_3_-2H-CO]^+^  192.1015[M−C_9_H_11_O_2_]^+^ 177. 0777[M−C_9_H_11_O_2_−CH_3_]^+^ | Tetrahydrojatrorrhizine |
| 9 | 3.37 | C_20_H_24_NO_4_ |  | 342.1693 | -2.052 | _ | 297[M-(CH_3_)_2_NH]^+^  265[M-(CH_3_)_2_NH-CH_3_OH]^+^  192[M-(CH_3_)_2_NH-CH_3_OH-CO-C_2_H_4_]^+^ | magnoﬂorine |
| 10 | 3.49 | C_17_H_20_O_9_ | 367.1032 | 369.1172 | -0.815 -2.173 | 735.2122[2M-H]^−^  193.0497[M-H-C_7_H_11_O_6_]^−^  191.0554[M-H-C_10_H_9_O_4_]^−^  173.0448[M-H-C_10_H_9_O_4_-H_2_O]^−^  147.0289[M-H-C_7_H_11_O_6_-CO_2_-2H]^−^ | _ | 4-O-feruloylquinic acid |
| 11 | 3.72 | C_17_H_20_O_9_ | 367.1032 | 369.1172 | -0.815 -2.173 | 735.2122[2M-H]^−^  193.0538[M-H-C_7_H_11_O_6_]^−^  191.0554[M-H-C_10_H_9_O_4_]^−^  175.0389[M-H-C_7_H_11_O_6_-H_2_O]^−^ 173.0448[M-H-C_10_H_9_O_4_-H_2_O]^−^ 147.0289[M-H-C_7_H_11_O_6_-CO_2_-CH_2_]− | _ | 5-O-Feruloylquinic acid |
| 12 | 3.83 | C_19_H_24_NO_3_ |  | 314.1745 | -3.501 | _ | 299.1514[M-CH_3_]^+^  283.1318[M-CH_4_-CH_3_]^+^  269.1169[M-CH_4_-CH_3_-CHO]^+^/[M-(CH_3_)_2_NH]^+^ | Magnocurarine |
| 13 | 3.97 | C_18_H_22_O_9_ | 381.1195 |  | 1.047 | 193.0498[M-H-C_7_H_11_O_6_]^−^  175.0391[M-H-C_7_H_11_O_6_-H_2_O]^−^ | _ | fernloylquinic acid methyl ester |
| 14 | 4.18 | C_19_H_24_NO_3_ |  | 314.1745 | -3.501 | _ | 299.1514[M-CH_3_]^+^  283.1318[M-CH_4_-CH_3_]^+^  269.1169[M-CH_4_-CH_3_-CHO]^+^/[M-(CH_3_)_2_NH]^+^ | Lotusine |
| 15 | 4.41 | C_21_H_26_NO_4_ |  | 356.1850 | -3.370 | _ | 311.0908[M-(CH_3_)_2_NH]^+^  279.1010[M-(CH_3_)_2_NH-CH_3_OH]^+^ | Menisperine |
| 16 | 4.68 | C_21_H_26_NO_4_ |  | 356.1849 | -3.650 | _ | 311.0908[M-(CH_3_)_2_NH]^+^  296.1042[M−4CH_3_]^+^  279.1010[M-(CH_3_)_2_NH-CH_3_OH]^+^ 264.0778[M-(CH_3_)_2_NH-CH_3_OH-CH_3_]^+^ | (+)N-Methylcorydine |
| 17 | 4.94 | C_21_H_26_NO_4_ |  | 356.1849 | -3.650 | _ | 311.1272[M-(CH_3_)_2_NH]^+^ 296.1039[M−4CH_3_]^+^  279.1013[M-(CH_3_)_2_NH-CH_3_OH]^+^ 264.0770[M-(CH_3_)_2_NH-CH_3_OH-CH_3_]^+^ 236.0835[M-(CH_3_)_2_NH-CH_3_OH-CH_3_]^+^ 192.1015[M-(CH_3_)_2_NH-CH_3_OH-CH_3_-CO-CH_4_]^+^ | Tetrahydropalmatine |
| 18 | 5.05 | C_26_H_34_O_11_ | 567.1780 |  | -5.802 | 521.2596[M-H]^-^ 341.1396[M-H-Glc-H_2_O]^-^ 329.1396[M -Glc-OCH_3_]^-^ | _ | (+/-)8-(4-Hydroxy-3-methoxyphenyl)-6,7-bis(hydroxymethyl)-3-methoxy-5,6,7,8-tetrahydro-2-naphthalenyl β-D-  glucopyranoside |
| 19 | 5.06 | C_20_H_26_NO_3_ |  | 328.1914 | 3.047 | _ | 283.1322[M-C_2_H_5_O]^+^ 269.1163[M-C_2_H_5_O-CH_3_]^+^ | Armepavine |
| 20 | 5.28 | C_19_H_18_NO_4_ |  | 324.1223 | -4.011 | _ | 309.0989[M-CH_3_]^+^ | demethylberberine |
| 21 | 5.32 | C_20_H_18_NO_5_ |  | 352.1172 | -3.692 | _ | 337.0936[M-CH_3_]^+^ 322.1155[M-2CH_3_]^+^ 308.1354[M-CH_3_-CO-H]^+^ | Oxoberberine |
| 22 | 5.48 | C_20_H_18_NO_5_ |  | 352.1172 | -3.692 | _ | 337.0936[M-CH_3_]^+^ 322.0706[M-2CH_3_]^+^ 308.0920[M-CH_3_-CO-H]^+^ | Oxoepiberberine |
| 23 | 5.49 | C_17_H_22_NO_4_ | 349.1525 |  | -1.973 | 334.0684[M+HCOO-CH_3_]^-^ 286.0726[M-H_2_O]^-^ 175.0390[M-H-CO]^-^ | _ | dasycarpamin |
| 24 | 5.51 | C_21_H_21_NO_5_ |  | 368.1488 | -1.089 | _ | 353.1247[M+H-CH_3_]^+^ 324.1225[M+H-CH_3_-CO-H]^+^ | hydroxylpalmatine |
| 25 | 5.52 | C_17_H_20_O_9_ | 367.1039 |  | 1.087 | 191.0553[M-H-C_7_H_11_O_6_]^−^  173.0446[M-H-C_10_H_9_O_4_-H_2_O]^−^ 149.0597[M-H-C_7_H_11_O_6_-CO_2_]^−^ 134.0362[M-H-C_7_H_11_O_6_-CO_2_-CH_3_]^−^ 111.0075[M-H-C_7_H_11_O_6_-2CO_2_-2H-  H_2_O]^-^ | _ | feruloylquinic acid |
| 26 | 5.54 | C_21_H_26_NO_4_ |  | 356.1849 | -3.650 | _ | 311.1258[M-(CH_3_)_2_NH]^+^  279.1010[M-(CH_3_)_2_NH-CH_3_OH]^+^ | Xanthoplanine |
| 27 | 5.67 | C_21_H_24_NO_4_ |  | 354.1689 | -4.518 | _ | 339.1080[MCH_3_]^+^  190.0859[M-2CH_3_-H]^+^ | N-Methyl canadine |
| 28 | 5.79 | C_20_H_20_NO_4_ |  | 338.1379 | -3.845 | _ | 323.1144[M-CH_3_]^+^ 308.0910[M-2CH_3_]^+^ 294.1117[M-2CH_3_-CO-H]^+^ | Columbamine |
| 29 | 5.88 | C_20_H_20_NO_4_ |  | 338.1362 | -8.872 | _ | 323.1095[M-CH_3_]^+^ 308.1235[M-2CH_3_]^+^ 280.2636[M-2CH_3_-CO]^+^ | Jatrorrhizine |
| 30 | 5.95 | C_19_H_15_NO_4_ |  | 322.1069 | -0.934 | _ | 307.0834[M-CH_3_]^+^ 292.0602[M-H-CO]^+^ 278.0809[M-CH_3_-CO]^+^ | berberrubine |
| 31 | 5.97 | C_22_H_28_NO_4_ |  | 370.2007 | -2.971 | _ | 206.1172[M-CH_3_-C_9_H_7_O_2_]^+^ | 1,2,9,10-Tetramethoxy-6,6-dimethyl-5,6,6a,7-tetrahydro-4H-dibenzo[de,g]quinolinium |
| 32 | 6.01 | C_20_H_18_NO_4_ |  | 336.1221 | -4.463 | _ | 321.0987[M-CH_3_]^+^ 306.0756[M-2CH_3_]^+^ 292.0960[M-CH_3_-H-CO]^+^ 278.0791[M-2CH_3_-CO]^+^ | Berberine |
| 33 | 6.07 | C_20_H_18_NO_4_ |  | 336.1221 | -4.463 | _ | 321.0986[M-CH_3_]^+^ 306.0759[M-2CH_3_]^+^ 292.0958[M-CH_3_-H-CO]^+^ 278.0771[M-2CH_3_-CO]^+^ | Isomer of berberine |
| 34 | 6.30 | C_19_H_34_O_15_ | 501.1841 |  | 3.186 | 457.1869[M-CO_2_]^-^ 413.1978[M-2CO_2_]^-^ 395.1863[M-2CO_2_-H_2_O]^-^ 371.1861[M-2CO_2_-H_2_O-CH_3_]^-^ | _ | γ-hydroxybutenolide deniatives II |
| 35 | 6.31 | C_20_H_18_NO_4_ |  | 336.1223 | -3.868 |  | 321.0988[M-CH_3_]^+^ 306.0759[M-2CH_3_]+ 292.0958[M-CH_3_-H-CO]+ 278.0783[M-2CH_3_-CO]+ | Isomer of berberine |
| 36 | 6.42 | C_19_H_18_O_11_ | 421.0759 |  | -4.028 | 377.1060[M-CO_2_]^-^  301.1446[M-C_4_H_8_O_4_]^-^ | _ | Mangiferin |
| 37 | 6.61 | C_26_H_30_O_9_ | 531.1849 |  | -4.731 | 485.1826 [M-H]^-^ 423.1819[M-H-H_2_O-CO_2_]^-^ 411.2003[M-H-H_2_O-C_2_O_2_]^-^ 327.2176[M-C_10_H_7_O_2_]^-^ | _ | Rutaevin |
| 38 | 6.63 | C_20_H_18_NO_4_ |  | 336.1224 | -3.570 | _ | 321.0988[M-CH_3_]^+^ 306.0765[M-2CH_3_]^+^ 292.0962[M-CH_3_-H-CO]^+^ | Isomer of berberine |
| 39 | 6.71 | C_18_H_34_O_5_ | 329.2333 |  | 0 | 311.2226[M-H-HO]^-^ 293.2119[M-H-2HO]^-^ 229.1442[M-H-HO-C_6_H_10_O]^-^  211.1334[M-H-CO_2_-(CH_2_)_4_CH_3_]^-^ 171.1018[M-H-OH-(CH_2_)_8_CH_3_OH]^-^ | _ | Sanleng acid |
| 40 | 7.05 | C_26_H_30_O_8_ | 470.1910 |  | -6.593 | 515.1920[M+HCOO]^-^ 469.1872[M -H]^-^ 425.2195[M -H-CO_2_]^-^ 409.2359[M -H-CO_2_-CH_4_]^-^ | _ | Obaculactone |
| 41 | 7.20 | C_21_H_22_NO_4_ | 352.1569 |  | 5.679 | _ | 337.0938[M-CH_3_]^+^ 308.0916[M-CH_4_-CO]^+^ 292.0981[M-4CH_3_]^+^ | Palmatine |
| 42 | 7.27 | C_31_H_46_O_5_ | 497.3378 |  | -4.816 | 423.1821[M-CO_2_-2H-CO]^-^ | _ | Poricoic acid A |
| 43 | 7.36 | C_16_H_18_O_9_ | 353.0871 |  | 8.960 | 193.0602[M-C_7_H_11_O_5_]- 191.0553[M-C_9_H_6_O_3_]- 173.0447[M-C_9_H_6_O_3_-H_2_O]- | _ | Cryptochlorogenic acid |
| 44 | 7.71 | C_13_H_16_O_10_ | 331.0658 |  | -3.915 | 313.2386[M-H-H_2_O]^-^ 168.0408[M-H-GLc]^-^ 149.0460[M-H-H_2_O-H]^-^ 125.0346[M-C_7_H_4_O_5_-2H_2_O]^-^ | _ | b-Glucogallin |
| 45 | 7.82 | C_26_H_30_O_7_ |  | 455.2051 | -2.862 | _ | 427.2062[M+H-CO]^+^ 409.2000[M+H-CO-H_2_O]^+^ | Obacunone |
| 46 | 7.84 | C_17_H_20_N_2_O | 313.1568 |  | 3.729 | 253.9937[M+HCOO-CH_3_]^-^ 148.0506[M+HCOO-CH_3_-C_7_H_8_N]^-^ | _ | Bis-[4-(dimethylamino)phenyl]methanone |
| 47 | 8.64 | C_18_H_32_O_2_ | 279.2312 |  | -6.423 | 265.2159[M-CH_2_]^-^ 232.1539[M-CH_2_-H_2_O-CH_3_]^-^ | _ | cis-9-cis-12-Linoleic acid |
| 48 | 9.18 | C_9_H_8_O_2_ |  | 149.0231 | -6.450 | _ | 131.0491[M-H_2_O]^+^ 103.0544[M-HCOOH]^+^ | cinnamic acid |
